# 

**Published:** 1904-09

**Authors:** 


PUBLISHED MONTHLY

### ATLANTA JOURNAL-RECORD OF MEDICINE.

Successor to Atlanta Medical and Surgical Journal, Established 1855, and Southern Medical Record, Established 1870.

VOL. VI. ATLANTA, GA., SEPTEMBER, 1904. No. 6.

